# A Longitudinal Multimodal Neuroimaging Study to Examine Relationships Between Resting State Glutamate and Task Related BOLD Response in Schizophrenia

**DOI:** 10.3389/fpsyt.2018.00632

**Published:** 2018-11-29

**Authors:** Elyse J. Cadena, David M. White, Nina V. Kraguljac, Meredith A. Reid, Jose O. Maximo, Eric A. Nelson, Brian A. Gawronski, Adrienne C. Lahti

**Affiliations:** ^1^Department of Psychiatry and Behavioral Neurobiology, University of Alabama at Birmingham, Birmingham, AL, United States; ^2^Magnetic Resonance Imaging Research Center, Auburn University at Birmingham, Birmingham, AL, United States

**Keywords:** schizophrenia, functional magnetic resonance imaging, magnetic resonance spectroscopy, glutamate, antipsychotic medication, cognitive task, anterior cingulate cortex

## Abstract

Previous studies have observed impairments in both brain function and neurometabolite levels in schizophrenia. In this study, we investigated the relationship between brain activity and neurochemistry in off-medication patients with schizophrenia and if this relationship is altered following antipsychotic medication by combining proton magnetic resonance spectroscopy (1H-MRS) with functional magnetic resonance imaging (fMRI). We used single voxel MRS acquired in the bilateral dorsal anterior cingulate cortex (ACC) and fMRI during performance of a Stroop color-naming task in 22 patients with schizophrenia (SZ), initially off-medication and after a 6-week course of risperidone, and 20 matched healthy controls (HC) twice, 6 weeks apart. We observed a significant decrease in ACC glutamate + glutamine (Glx)/Creatine (Cr) levels in medicated SZ patients compared to HC but not compared to their off-medication baseline. In off-medication SZ, the relationship between ACC Glx/Cr levels and the blood oxygen level-dependent (BOLD) response in regions of the salience network (SN) and posterior default mode network (DMN) was opposite than of HC. After 6 weeks, the relationship between Glx and the BOLD response was still opposite between the groups; however for both groups the direction of the relationship changed from baseline to week 6. These results suggest a mechanism whereby alterations in the relationship between cortical glutamate and BOLD response is disrupting the modulation of major neural networks subserving cognitive processes, potentially affecting cognition. While these relationships appear to normalize with treatment in patients, the interpretations of the results are confounded by significant group differences in Glx levels, as well as the variability of the relationship between Glx and BOLD response in HC over time, which may be driven by factors including habituation to task or scanner environment.

## Introduction

Schizophrenia is a disorder characterized by cognitive impairment. The anterior cingulate cortex (ACC) has been identified as being involved in cognitive processes, including mediating executive functioning and conflict monitoring. Functional magnetic resonance (fMRI) studies have shown alterations in ACC function and its associated cognitive network during cognitive processes (1–4). Proton magnetic resonance spectroscopy (1H-MRS) allows for *in vivo* measurement of brain metabolites such as glutamate, an amino acid involved in excitatory neurotransmission (5) and metabolism (6, 7). There is increasing evidence suggesting that abnormal functioning in the glutamatergic system may influence the pathogenesis of schizophrenia (8–11). Previous 1H-MRS schizophrenia studies report abnormal glutamatergic levels (12–17) that appear to vary depending on voxel location, illness stage, and medication status (18). Studies in the ACC have reported increased glutamate (or the combination of glutamate and glutamine, Glx) levels in medication naïve (19) and minimally treated patients (20) and decreased levels in medicated first episode (21) and chronic patients with schizophrenia (15, 22, 23).

Using functional MRI (fMRI), several large-scale brain networks have been identified and defined in terms of the processes they are thought to subserve (24, 25). Of particular interest are the salience network (SN) and posterior default mode network (DMN). The SN which comprises the anterior insula and dorsal ACC is involved in the detection and processing of salient events (26) as well as switching between cognitive networks and the DMN (27). The posterior DMN is comprised of a set of regions (precuneus, inferior parietal gyrus, hippocampus, posterior cingulate (PCC), and medial prefrontal gyrus) that are active during rest and internal referencing tasks and deactivate during external goal-driven tasks (24, 28, 29). A balance between the activation of task positive networks and the deactivation of the DMN appears necessary for cognitive functioning (30). Previous fMRI studies report disrupted task-state and resting-state blood-oxygen level-dependent (BOLD) signal in the SN and posterior DMN and the networks' relationship with other networks in schizophrenia (31–34). These studies strongly suggest that brain activity in the SN and posterior DMN is altered in patients with schizophrenia.

Brain glutamate metabolism has been linked to the fMRI BOLD signal response (35). Glutamate is strongly involved in the brain's energy turnover as the majority of resting energy consumption in the awake brain is tightly coupled with neuronal activity (36). Indeed, some MRS-fMRI studies have reported evidence for a positive correlation between glutamate and the BOLD response both within the measured region as well as within distant regions from the source region, suggesting that glutamate is also related to long-range connections between regions (37). Combining MRS-fMRI analyses, especially when brain function is evaluated in terms of neural networks, may provide additional insights into the relationship between glutamate and neuronal response during resting state or task performance (38–40). In addition, combining MRS with fMRI in the same subjects, rather that measuring a single variable in isolation, allows us to concurrently investigate several (possibly connected) mechanisms involved in schizophrenia which may contribute to a better understanding of the disease mechanisms. Previous studies reported significant correlations between Glx/Cr levels and BOLD signal in healthy controls that was not present or reversed in medicated patients with schizophrenia (41, 42). Importantly, two studies identified a negative relationship between ACC glutamate and the BOLD response in the posterior DMN in healthy controls and this relationship was found to be opposite in patients with schizophrenia (40, 43).

However, the majority of combined MRS-fMRI schizophrenia studies enrolled patients treated with antipsychotic drugs (APD), all dopamine (DA) receptor antagonists. Because the neural basis of cognitive control relies on fronto-cortical-striatal circuitry known to be under DA modulation (44, 45), modulation of cognitive control functional activity with DA antagonists is expected. Indeed, APDs have been shown to affect brain function (46, 47) In addition, as stated previously, glutamate levels have been shown to be affected by APD treatment (18). Therefore, it is important to investigate the abnormalities in patients with schizophrenia prior to APD exposure.

The purpose of this study was to investigate the relationship between brain function and neurochemistry of the ACC in off-medication patients with schizophrenia and matched healthy controls using 1H-MRS and fMRI during performance of a cognitive control task, as well as evaluate the effect of antipsychotic treatment on these measures and their relationships.

Based on prior findings, we hypothesized that Glx levels would be reduced in patients with schizophrenia following 6 weeks of treatment. We also hypothesized to find a significant relationship between ACC Glx levels and BOLD signal in the ACC and regions of the posterior DMN in healthy controls that would be altered in off-medication patients (40). Finally, to the extent that we expected a change in glutamate level with treatment, we also hypothesized that, following 6 weeks of APD, patients with schizophrenia would present a relationship between Glx and the ACC and posterior DMN BOLD signal more reflective of healthy controls.

## Materials and methods

### Subjects

Twenty-eight subjects with schizophrenia and schizoaffective disorder (SZ) were recruited from the outpatient psychiatry clinics and emergency room at the University of Alabama at Birmingham to participate in the study based on being off antipsychotic medication for at least 10 days (medication was not discontinued to meet this criterion). None of the patients had been on depot medication prior to discontinuing medication. Twenty-five healthy control subjects (HC), without personal or family history in a first-degree relative of significant DSM-IV-TR Axis I disorders were recruited using advertisement in flyers and the university's newspaper. Exclusion criteria were major medical conditions, substance abuse or dependence (except for nicotine) within 6 months of imaging, previous head injury, a neurologic disorder, loss of consciousness for more than 2 min, and pregnancy. The Institutional Review Board of the University of Alabama at Birmingham provided approval for the study and all subjects gave written informed consent prior to participating. Before signing consent, each subject with schizophrenia completed an Evaluation to Sign Consent Form (48).

Diagnoses were established using subjects' medical records and the Diagnostic Interview for Genetic Studies (DIGS) (49). General cognitive function for each subject was characterized by the Repeatable Battery for the Assessment of Neuropsychological Status (RBANS) (50).

Patients were scanned while off-medication, and then entered into a 6-week trial with risperidone (flexible dosing regimen), at the end of which they received a second scan. Symptom severity was assessed with the Brief Psychiatric Rating Scale (BPRS) (51) and its positive and negative subscales. Medication compliance was monitored by pill counts. HC were scanned twice at 6-week intervals. Prior to each scanning session, all subjects underwent a urine drug screen.

### Controlling for movement

Four SZ and two HC subjects were excluded due to movement during scanning. Subjects were excluded when the motion parameters showed >2 mm translation or 2° rotation within a run. A linear mixed model of the mean scan-to-scan head movement for the six movement parameters (linear movement in the x, y, and z axes and rotational movement of pitch, roll, and yaw) indicated no significant differences for group (HC vs. SZ), time (baseline vs. week 6), or group × time (See Supplement Table 1). Two SZ subjects and three HC were excluded for lack of complete task data/performance. A total of 22 SZ and 20 HC subjects remained in the off-medication baseline analyses. Two SZ subjects did not perform the task at the 6-week scan, leaving 20 SZ, and 20 HC subjects at the 6-week analyses (Table 1).

**Table 1:** Demographics, clinical, and behavioral measures^a^.

	**SZ (*****n*** = **22)**		**HC (*****n*** = **20)**		**t/x^2^**	***P*-value**
Age, years	33 (9.78)		33.05 (9.31)		−0.002	0.99
Sex, M/F	17/5		14/6		0.63	0.54
Parent SES^b^	7.89 (5.85)		5.68 (3.92)		1.34	0.19
Smoking Status (Smoker/Non-smoker)	19/3		10/10		1.72	0.06
Smoking, packs per day	0.73 (0.54)		0.39 (0.57)		1.97	0.06
Medication naïve	*n* = 9				
Months off medication	27.75 (49.99)				
Diagnosis (Schizophrenia/schizoaffective)	(19/3)				
Age of onset, years	21.86 (3.38)				
RBANS Total^c^	70.55 (12.67)		93.5 (14.81)		−2.89	0.006
	**SZ 0**	**SZ 6**^d^	**HC 0**	**HC 6**	
**BPRS**^e^						
Total	48.59 (10.32)	29.52 (8.14)			8.88	< 0.001
Positive	8.86 (2.48)	4.52 (2.58)			7.82	< 0.001
Negative	7.05 (2.38)	5.14 (2.31)			3.01	0.007
**ACC Glx/Cr**	0.67 (0.07)	0.68 (0.05)	0.70 (0.07)	0.72 (0.07)	
Group						0.047
Time						0.183
Interaction						0.895
**TASK REACTION TIME**, **SEC**						
Congruent	0.91 (0.18)	0.90 (0.19)	0.80 (0.10)	0.79 (0.13)	
Incongruent	1.04 (0.18)	1.03 (0.23)	1.00 (0.14)	0.91 (0.10)	
Stroop	0.13 (0.07)	0.13 (0.07)	0.19 (0.09)	0.14 (0.08)	
**MISSING TRIALS**						
Congruent	8.45 (11.86)	6.80 (13.76)	2.85 (8.38)	2.75 (5.30)	
Incongruent	4.32 (7.08)	4.20 (7.85)	1.05 (2.63)	1.25 (2.61)	
**TASK ERRORS**						
Congruent	10.71 (15.83)	8.06 (12.49)	2.75 (4.28)	4.30 (7.14)	
Incongruent	4.52 (5.23)	3.28 (3.75)	4.25 (5.46)	3.90 (5.09)	

*SZ, schizophrenia; HC, healthy control. SZ 0, off-medication baseline schizophrenia; SZ 6, 6 weeks medicated schizophrenia. HC 0, healthy controls baseline; HC 6, healthy controls 6 weeks. ACC, Anterior cingulate cortex; Glx/Cr, Glutamate + Glutamine/Creatine*.

a *Mean (SD) unless indicated otherwise*.

b *Ranks determined from Diagnostic Interview for Genetic Studies (1–18 scale); higher rank (lower numerical value) corresponds to higher socioeconomic status; data not available for 4 SZ subjects*.

c *Repeatable Battery for Neuropsychological Status. Data not available for 5 SZ subjects*.

d *n = 20*.

e *Brief Psychiatry Rating Scale (1–7 scale); positive (conceptual disorganization, hallucinatory behavior, and unusual thought content); negative (emotional withdrawal, motor retardation, and blunted affect); data not available for 1 SZ subject*.

### Functional task

Subjects performed a computerized version of the Stroop color-naming task (52). Stimuli consisted of three words: “RED,” “GREEN,” or “BLUE,” displayed in one of the corresponding colors. Trials were designated as either “congruent” or “incongruent,” where incongruent trials reflected a difference between the word and the color of the word. Subjects were instructed to indicate the color and ignore the word. They were instructed to respond as quickly and as accurately as possible and responses were recorded by button press. An IFIS-SA system (*in vivo*, Orlando, Florida) running E-Prime software (version 1.2; Psychology Software Tools, Pittsburgh, Pennsylvania) was used to control stimulus delivery and record responses and reaction times. The event-related design consisted of three runs of 88 trials per run (~30% incongruent, 70% congruent; to increase conflict effect, the numbers of incongruent trials were less than congruent trials). The 3 s trials were comprised of a word stimulus for 1.5 s and a fixation cross for 1.5 s. Both SZ and HC completed a baseline session and a second session, 6 weeks later, corresponding to pre and post antipsychotic medication in SZ. All participants completed a practice run in the laboratory before each scanning session.

### Image acquisition

All imaging was performed on a 3T head-only MRI scanner (Magnetom Allegra, Siemens Medical Solutions, Erlangen, Germany), with a circularly polarized transmit/receive head coil. MRS sequences were always acquired prior to the functional ones.

### MRS

A series of sagittal, coronal, and axial T1-weighted anatomical scans serving as MRS-localizers were acquired for spectroscopic voxel placement. Slices were aligned to anatomical midline to control for head tilt. The MRS voxel was placed in a region of the bilateral dorsal ACC on the basis of the sagittal and coronal images. Manual shimming was performed to optimize field homogeneity across the voxel, and chemical shift selective (CHESS) pulses were used to suppress the water signal. Spectra were acquired using the point-resolved spectroscopy sequence (53) (PRESS; TR/TE = 2,000/80 ms to optimize the glutamate signal, number of averages = 256, voxel size 2.7 × 2 × 1 cm^3^). All of the 22 off-medication baseline patients with schizophrenia that completed the Stroop task also had ACC Glx measurements. Of the 20 patients with schizophrenia with 6 weeks medication that completed the Stroop task, 20 had ACC Glx measurements. Of the 20 HC, 20 had ACC Glx measurements at baseline and 19 at 6 weeks.

### fMRI

fMRI data were acquired using the gradient recalled echo-planar imaging (EPI) sequence (repetition time/echo time [TR/TE] = 2,100/30 ms, flip angle = 70°, field of view = 24 × 24 cm^2^, 64 × 64 matrix, 4-mm slice thickness, 1-mm gap, 26 axial slices). A high-resolution structural scan was acquired for anatomical reference using the three-dimensional T1-weighted magnetization prepared rapid acquisition gradient-echo sequence (TR/TE/inversion time [TI] = 2,300/3.93/1,100 ms, flip angle = 12°, 256 × 256 matrix, 1-mm isotropic voxels).

### Statistical analyses

#### Behavior and demographics

Analyses were conducted using SPSS 20 (IBM SPSS Inc., Chicago, IL). Group comparisons were performed using chi-square or analysis of variance, as appropriate. Analyses of reaction time (RT) for correct trials [congruent, incongruent, and Stroop (incongruent—congruent)] and errors (congruent, incongruent) were analyzed using linear mixed models comparing fixed effects of group (HC vs. SZ), time (off-medication vs. week six), condition (congruent vs. incongruent), and interactions. *Post-hoc* analyses were performed where appropriate with Bonferroni correction.

#### Stroop bold fMRI

Data analyses were implemented in SPM8 (Wellcome Trust Centre for Neuroimaging). Preprocessing included slice-timing correction, realignment, reslicing to 1.5 mm isotropic voxels, motion/artifact correction using ArtRepair (54), DARTEL normalization, and smoothing (4 mm full width at half maximum Gaussian kernel). Analysis for the Stroop task consisted of a single-subject voxel-by-voxel general linear model. Five conditions were included: incongruent trials, congruent trials, stimulus repetitions [exact repetition of a previous trial (55)], error, and no response trials. The conditions were convolved with the canonical hemodynamic response function with temporal derivatives. The contrast of interest was correct incongruent trials minus correct congruent trials, subsequently referred to as the Stroop effect. A contrast z-map of the BOLD signal during the Stroop effect was generated for each participant at each time point.

#### MRS

MRS data were analyzed in jMRUI (version 3.0) (56). The residual water peak was removed using the Hankel-Lanczos singular values decomposition (HLSVD) filter (57). Spectra were quantified in the time domain using the AMARES (advanced method for accurate, robust, and efficient spectral fitting) algorithm (58). Prior knowledge derived from *in vitro* and *in vivo* metabolite spectra was included in the model. A phantom solution of 20 mM glutamate in buffer was imaged using the MRS parameters from the *in vivo* study. The resulting spectrum was quantified in jMRUI, and this model was used to fit the *in vivo* data. The model consisted of peaks for NAA, choline (Cho), creatine (Cr), and 3 peaks for glutamate + glutamine (Glx), which correspond to the H-4 resonance of Glu. Amplitude, line width, and chemical shift were optimized for each peak. Cramer-Rao lower bounds (CRLB) (59–61) were calculated for each peak. Exclusion criteria were CRLB >20% or failure of the fitting algorithm. NAA, Glx, and Cho were quantified with respect to Cr and compared across groups using one-way ANOVA with an alpha level of 0.05.

#### Behavior/MRS correlations

The relationships between glutamate levels, Stroop task behaviors, RBANS total index, and BPRS positive and negative subscales were analyzed by Pearson correlation (see in Supplement Results).

#### fMRI/MRS relationship

ACC Glx/Cr levels were included as a regressor in the Stroop BOLD activation for each corresponding participant at both groups and both timepoints, separately (e.g., baseline HC ACC Glx/Cr correlation with baseline HC Stroop BOLD signal). A multiple regression analysis was used to test for an interaction between the BOLD Stroop effect Group (HC vs. SZ) and Glx. Tests for voxels where the relationship between the BOLD Stroop effect and Glx between groups (HC vs. SZ) were made using two-sample *t*-tests at both time points separately (baseline; 6 weeks) at *p* < 0.05.

fMRI analyses were corrected for multiple comparisons using small-volume correction (SVC) in accordance with Gaussian random field theory (*p* < 0.05). In order to limit observable findings to the networks of interest, results were restricted to two separate inclusive masks containing regions that comprise the (1) SN and (2) posterior DMN. The SN mask contained the bilateral insula and ACC and the posterior DMN mask contained the bilateral PCC, precuneus, and inferior parietal lobule, and hippocampus from the automated anatomical labeling (AAL) atlas in the WFU Pickatlas (62). For illustration purposes, the signal was extracted from significant regions using REX (CIBSR Stanford University, CA) with a 6 mm ROI and the extracted first eigenvariate signal was then plotted against each participant's associated ACC Glx/Cr value.

## Results

### Demographics and behavior

No significant differences were observed between HC and SZ for matching criteria: age, gender, parental occupation, or smoking packs per day. At the time of baseline fMRI acquisition, SZ had been off antipsychotic medication for a mean of 832 days, median: 240 days. Mean baseline BPRS positive subscale score of 8.8 ± 2.5 indicated a high burden of psychotic symptoms in patients. Over the course of 6 weeks of risperidone SZ demonstrated a significant reduction in symptoms as indicated by the BPRS. Risperidone dose at the end of the study was 4.17 ± 1.92 mg. Patients also received the following psychotropic medications: benztropine (*n* = 11), trazodone (*n* = 2), divalproex sodium (*n* = 1), and amitriptyline (*n* = 1).

Reaction Times (RTs) for correct responses are presented in Table 1. There was a significant effect of Group (HC vs. SZ), *F*_(1, 40)_ = 4.47, *p* < 0.05, Condition (Incongruent vs. Congruent), *F*_(1, 116)_ = 122.88, *p* < 0.001, and Group × Time interaction, *F*_(1, 116)_ = 4.00, *p* < 0.05. *Post-hoc* analyses demonstrated both HC and SZ had faster reaction times during congruent trials than incongruent trials (*p* < 0.01), demonstrating the Stroop effect as expected. HC had significantly faster reaction times than off-medication SZ during congruent trials, and significantly faster reaction times than medicated SZ during both congruent and incongruent trials. No significant differences in reaction times were observed within SZ between off-medication baseline and week 6 of risperidone. Task related behavioral measures are presented in Table 1. No significant differences were observed for Group, Time, or interactions.

### fMRI

Results of Stroop task-based BOLD fMRI signal in off-medication SZ patients and the longitudinal differences after 6 weeks of medication have been previously published (63). In the SN, off-medication SZ showed significantly less BOLD response in the ACC and insula compared to baseline HC during Stroop task performance. In the posterior DMN, off-medication SZ showed significantly less BOLD response in the PCC, inferior parietal, precuneus, and hippocampus compared to baseline HC. Inspection of group deactivation patterns indicated that there was limited deactivation in the posterior DMN in both HC and SZ at both time points during the Stroop task than would be expected (Supplement Figure 1).

### MRS

MRS results for the HC and SZ groups are presented in Table 1. ACC Glx/Cr showed a significant effect of group [*F*_1, 40_ = 4.25, *p* < 0.05]. Medicated SZ showed decreased ACC Glx/Cr relative to HC at week 6 (*p* < 0.05). There was no significant group difference at baseline.

### Combined fMRI and MRS

#### Off-medication/baseline

**Figure 1:**
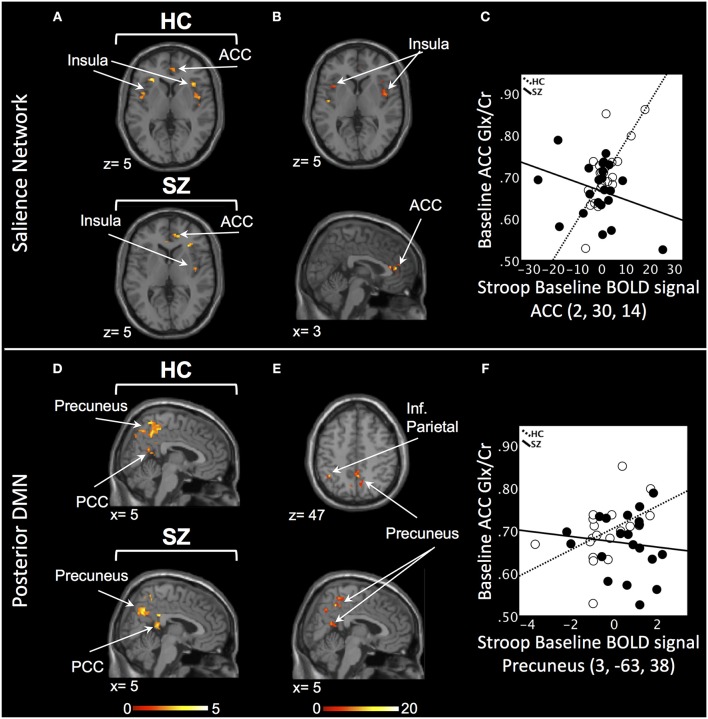
Baseline relationship between ACC Glx and Stroop BOLD signal. **(A)** In the salience network, relationship between Glx levels and Stroop BOLD signal in healthy controls (HC) and off-medication patients with schizophrenia (SZ) in the ACC and insula. **(B)** Baseline BOLD Stroop group × ACC Glx interaction significant in bilateral insula and ACC in the salience network. **(C)** Descriptive plot of ACC Glx and ACC Stroop BOLD signal (MNI coordinates 2, 30, 14) reflecting the significant positive correlation in HC and the lack of correlation in off-medication SZ. **(D)** In the posterior default mode network (DMN), relationship between Glx levels and Stroop BOLD signal in HC and off-medication SZ in the precuneus, posterior cingulate cortex (PCC) and inferior parietal lobule (Inf. Parietal). **(E)** Baseline BOLD Stroop group × ACC Glx interaction significant in the precuneus and inferior parietal lobule in the posterior DMN. **(F)** Descriptive plot of ACC Glx and Precuneus Stroop BOLD signal (MNI coordinates 3, −63, 38) reflecting the significant positive correlation in HC and the lack of correlation in off-medication SZ. x and z coordinates refer to Montreal Neurological Institute (MNI) space. Color bar on bottom indicates t-score. All analyses were thresholded at *P*_SVC_ < 0.05.

In the SN, there was a relationship between ACC Glx and Stroop BOLD signal in right ACC and bilateral insula in baseline HC, and in bilateral ACC and right insula in off-medication SZ (*P*_SVC_ < 0.05, Figure 1A; Supplement Table 2). In each group, the signals extracted from the most significant ACC and insula clusters were plotted against each other. In both group, the signals from these regions were highly correlated (HC: *r* = 0.86; off-medication SZ: *r* = 0.73). Significant Stroop BOLD group × Glx interactions were observed in the bilateral ACC and insula (*P*_SVC_ < 0.05, Figure 1B). A descriptive plot of the extracted interaction signal from the ACC shows a significant positive correlation between ACC Glx and ACC BOLD signal in baseline HC that was not observed in the off-medication SZ group (Figure 1C). The between-group analysis revealed significant group differences within bilateral insula and left ACC (*P*_SVC_ < 0.05, Table 2).

**Table 2:** Differences in the relationship between anterior cingulate cortex (ACC) Glx and the Stroop BOLD signal in schizophrenia compared to healthy controls.

**Region**	**Hemisphere**	**x, y, z**	**Voxels**	**Peak *t*-value**
**BASELINE**				
**Salience Network**				
**HC vs. SZ**			
Cluster 1 (ACC)	L	−8, 31, 18	1069	4.94
Cluster 2 (Insula)	L	−30, 25, −7	110	3.83
Cluster 3 (Insula)	R	43, 18, 2	346	3.69
Cluster 4 (Insula)	L	−36, 0, −9	126	3.49
Cluster 5 (Insula)	L	−42, 9, −7	96	3.29
Cluster 6 (Insula)	R	42, −15, −3	111	3.16
Cluster 7 (ACC)	L	−4, 1, 29	75	2.62
**Posterior DMN**				
**HC vs. SZ**			
Cluster 1 (Inf. Parietal)	R	36, −47, 53	258	4.05
Cluster 2 (Inf. Parietal)	L	−28, −45, 47	379	3.98
Cluster 3 (Precuneus)	R	8, −63, 56	1184	3.97
Cluster 4 (PCC)	R	4, −36, 29	54	3.79
Cluster 5 (Inf. Parietal)	R	45, −41, 45	76	3.11
Cluster 6 (Precuneus)	L	−8, −66, 36	99	3.10
Cluster 7 (Hippocampus)	R	26, −38, 2	49	2.95
**6 WEEKS**				
**Salience Network**				
**HC vs. SZ**			
Cluster 1 (ACC)	R	0, 6, 26	66	3.59
Cluster 2 (Insula)	L	−36, 10, −2	241	3.44
Cluster 3 (ACC)	R	3, 39, 21	77	3.26
Cluster 4 (Insula)	R	43, 15, −6	481	3.25
Cluster 5 (Insula)	R	36, −19, 9	85	3.04
Cluster 6 (ACC)	L	−9, 30, 22	87	2.93
Cluster 7 (Insula)	R	33, 16, 7	54	2.90
Cluster 8 (Insula)	L	−37, −4, 12	112	2.75
Cluster 9 (ACC)	R	6, 27, 21	70	2.68
**Posterior DMN**				
**HC vs. SZ**			
Cluster 1 (Inf. Parietal)	L	−38, −59, 45	592	4.29
Cluster 2 (Precuneus)	L	−12, −72, 34	606	3.41
Cluster 3 (Inf. Parietal)	R	38, −45, 45	105	3.25
Cluster 4 (Hippocampus)	R	21, −33, −2	73	3.11
Cluster 5 (Inf. Parietal)	R	40, −38, 51	51	3.09
Cluster 6 (Precuneus)	R	6, −55, 54	125	2.58
Cluster 7 (PCC)	R	9, −40, 21	66	2.53
Cluster 8 (Precuneus)	R	10, −51, 22	61	2.50
Cluster 9 (Precuneus)	L	−4, −48, 61	125	2.41
Cluster 10 (Inf. Parietal)	R	27, −55, 49	68	2.35
Cluster 11 (Hippocampus)	L	−33, −30, −12	95	3.57
Cluster 12 (Precuneus)	L	−6, −54, 33	62	2.86

*HC, Healthy Control; SZ, schizophrenia; L, left; R, right; ACC, anterior cingulate cortex; Inf. Parietal, inferior parietal cortex; PCC, posterior cingulate cortex; DMN, default mode network. x, y, z, refer to Montreal Neurological Institute coordinates*.

In the posterior DMN, there was a relationship between ACC Glx and Stroop BOLD signal in bilateral hippocampus, precuneus, inferior parietal lobule, and PCC in both groups (Figure 1D; Supplement Table 2). Significant Stroop BOLD group × Glx interactions were observed in the precuneus and inferior parietal lobule (*P*_SVC_ < 0.05, Figure 1E). A descriptive plot of the extracted interaction signal from the precuneus shows a significant positive correlation between ACC Glx and BOLD signal in the precuneus in baseline HC that was not observed in the off-medication SZ group (Figure 1F). The between-group analysis revealed significant group differences within bilateral inferior parietal lobule, precuneus, right hippocampus and right PCC (*P*_SVC_ < 0.05, Table 2).

#### 6 Weeks

**Figure 2:**
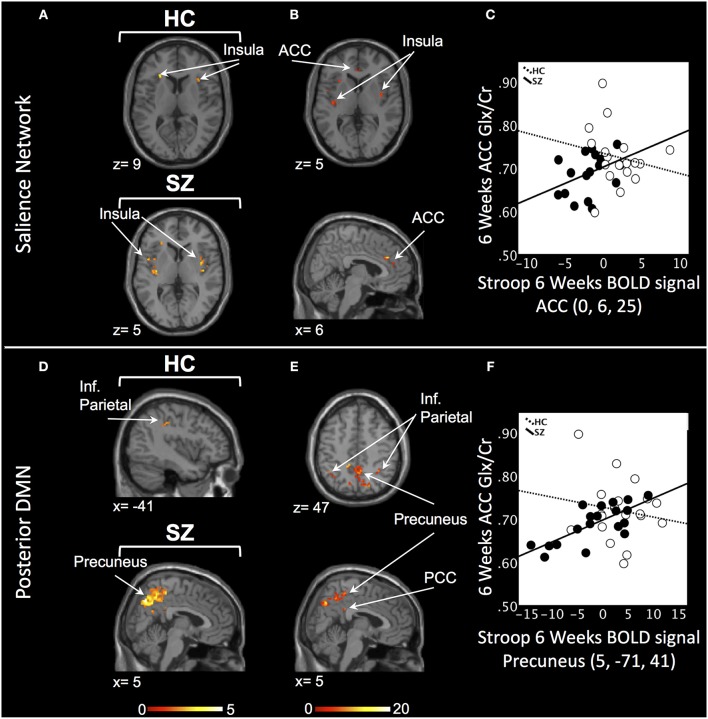
Six weeks relationship between ACC Glx and Stroop BOLD signal. **(A)** In the salience network, relationship between Glx levels and Stroop BOLD signal in the insula in healthy controls (HC) and in the ACC and insula in medicated patients with schizophrenia (SZ). **(B)** Six weeks BOLD Stroop group × ACC Glx interaction significant in bilateral insula and ACC in the salience network. **(C)** Descriptive plot of ACC Glx and ACC Stroop BOLD signal (MNI coordinates 0, 6, 25) reflecting the significant positive correlation in medicated SZ and the lack of correlation in HC. **(D)** In the posterior default mode network (DMN), relationship between Glx levels and Stroop BOLD signal in the inferior parietal lobule (Inf. Parietal) in HC and in the precuneus, inferior parietal lobule in medicated SZ. **(E)** Six weeks BOLD Stroop group × ACC Glx interaction significant in the precuneus, PCC, and inferior parietal lobule in the posterior DMN. **(F)** Descriptive plot of CC Glx and Precuneus Stroop BOLD signal (MNI coordinates 5, −71, 41) reflecting the significant positive correlation in medicated SZ and the lack of correlation in HC. x and z coordinates refer to Montreal Neurological Institute (MNI) space. Color bar on bottom indicates t-score. All analyses were thresholded at *P*_SVC_ < 0.05.

In the SN, there was a relationship between ACC Glx and Stroop BOLD signal in bilateral insula in HC and, in medicated SZ in bilateral insula and bilateral ACC (*P*_SVC_ < 0.05, Figure 2A; Supplement Table 3). In medicated SZ the signals extracted from the most significant ACC and insula clusters were highly correlated (medicated SZ: *r* = 0.96). Significant Stroop BOLD group × Glx interactions were observed in the ACC and insula (*P*_SVC_ < 0.05, Figure 2B). A descriptive plot of the extracted interaction signal from the ACC shows a significant positive correlation between ACC Glx and ACC BOLD signal in medicated SZ that was not observed in the HC group at week 6 (Figure 2C). The between-group analysis revealed significant group differences within bilateral insula and ACC (*P*_SVC_ < 0.05, Table 2).

In the posterior DMN, there was a ACC Glx and Stroop BOLD signal relationship in the left inferior parietal cortex in HC and bilateral hippocampus, inferior parietal lobule, and right precuneus in medicated SZ (Figure 2D; Supplement Table 3). Significant Stroop BOLD group × Glx interactions were observed in the precuneus, PCC, and inferior parietal lobule (*P*_SVC_ < 0.05, Figure 2E). A descriptive plot of the extracted interaction signal from the precuneus shows a significant positive correlation between ACC Glx and precuneus BOLD signal in medicated SZ that was not observed in HC at week 6 (Figure 2F). The between-group analysis revealed significant group differences within bilateral precuneus, inferior parietal lobule, right hippocampus, and right posterior cingulate (*P*_SVC_ < 0.05, Table 2).

## Discussion

The current study combined fMRI and 1H-MRS to investigate the relationship between ACC Glx and task-based BOLD signal in off-medication patients with schizophrenia and after 6 weeks of medication. The main findings are: (1) A significant decrease in ACC Glx levels in medicated SZ patients compared to HC but not compared to their off-medication baseline. (2) In off-medication SZ, the relationship between ACC Glx and the BOLD response in regions of the SN and posterior DMN was opposite than that of HC. (3) After 6 weeks, these relationships were still opposite between the groups; however for both groups the direction of the relationship had changed from baseline to week 6.

Over 6 weeks of treatment, we observed a significant decrease in ACC Glx compared to HC, but not compared to their off-medication baseline. There are a limited number of longitudinal studies evaluating the effect of short-term antipsychotic treatment on glutamatergic metabolites. In medication-naïve/ minimally treated first episode psychosis patients, Egerton reported a reduction in ACC glutamate after 4 weeks of treatment with amisulpride (64). In chronic patients washed out of medications, Szulc reported a decrease in temporal lobe Glx following 4 weeks of treatment with a variety of antipsychotic medications (65). In medication-naïve FEP subjects compared to healthy controls, de la Fuente-Sandoval observed higher baseline striatal glutamate and a significant reduction in striatal glutamate after 4 weeks of risperidone treatment (66). We acknowledge that the size of our sample was limited and there is a need to address this question in the future with larger sample size.

In HC, at baseline, interindividual differences in ACC Glx predicted the strength of the ACC BOLD response; those with higher ACC Glx levels showed greater ACC BOLD response, suggesting local resting state neurochemical concentrations are modulating local neural activity generated during task performance. We also identified a correlation between ACC Glx and the BOLD response in the insula, bilaterally; these results are in agreement with those of Duncan who identified a similar correlation between ACC glutamate and insula BOLD response during task performance (67). Suggestive of a network basis for concomitant identification of the ACC and the insula in this analysis, we identified strong correlations (*r* > 0.7) between the signal extracted from the ACC and the signal extracted from the insula. In addition, we found that ACC Glx levels were also positively and significantly correlated with the BOLD response in regions of the posterior DMN. These results are in contrast to two prior studies (40, 43) where ACC Glx was found to *negatively* correlate with the BOLD response in posterior DMN: higher levels of Glx measured in the ACC predicted greater deactivation of the DMN during task performance. Inspection of our BOLD data indicates that there was a limited deactivation of the DMN during Stroop performance in the region of the precuneus (see Supplement Figure 1); this could be explained by differences in task difficulty between our and the before mentioned studies (68) and, indeed, task difficulty has been shown to alter the relationship between ACC Glx and posterior DMN BOLD (69). There have been now a number of studies demonstrating correlations between glutamate and the BOLD signal in regions outside that in which glutamate is measured (40–42, 67, 69). It can be argued that local neurometabolites are likely to contribute to the activity of distant projections areas; this likely involves complex synaptic transmission where a number of neurotransmitters, including glutamate, tune the neuronal projections and thus affect the BOLD signal in distant regions. Known glutamatergic projections between the ACC and the insula and posterior DMN are hypothesized to support this modulation (70).

The group by Glx by BOLD response analysis identified significant interactions in the relationship between Glx and BOLD in regions of the ACC, insula, and posterior DMN. In these regions, off-medication SZ showed an altered pattern compared to HC: higher ACC Glx predicted lower BOLD response. Importantly, these alterations were found in the absence of a group differences in Glx levels. Like Falkenberg and Overbeek, we observed an opposite relationship between ACC Glx and regions of the posterior DMN between the groups; however in contrast to them, the relationship was in the opposite direction. Again, this discrepancy might be explained by the limited deactivation in the DMN seen in our study. Another major difference between this and the other studies is that our patients were off-medication. Together, our results suggest alterations in the relationship between Glx and the BOLD response locally, in the ACC, as well as in the long-range connections between the ACC, the insula, and the posterior DMN that are modulated by glutamate. Group differences might emerge as a consequence of altered overall regional ratio of excitation over inhibition, modulated by a number of neurotransmitters, including glutamate and GABA (71), as well as of known abnormal functional and structural connectivity between these brain regions (34, 72).

After 6 weeks, the group by Glx by BOLD analysis identified again significant group interactions in regions of the ACC, insula, and posterior DMN. However, in these regions, the relationships between Glx and BOLD response were now positive in medicated SZ and negative in HC. This is a complex picture as both SZ and HC show differences in the direction of the relationship between baseline and week 6. Thus, this effect cannot be attributed to an effect of medication alone. To complicate results' interpretation, medicated SZ had significantly lower level of Glx compared to HC; in addition, because of habituation, it is possible that the task became easier to perform the second time, and both Glx levels and task difficulty have been showed to alter the relationship between ACC Glx and posterior DMN BOLD (69).

### Strengths and limitations

To avoid confounding medication effects and minimize data variance, we only enrolled off-medication SZ, matched groups on several key factors, and used a rigorous longitudinal design with a single antipsychotic medication. Also, we attempted to control for the effect of time by scanning a HC group 6 weeks apart. Supporting the significance of controlling for time, a component lacking in many studies, our results indicate variability of the relationship between Glx and BOLD response in HC over time, which may be driven by factors including habituation to task or scanner environment. This combined MRS-fMRI study obtained neurometabolite levels that, given the spectroscopy sequence in the 3T MRI scanner, were unable to distinguish the overlapping glutamate, glutamine, and GABA peaks from each other. As studies have shown differences in these metabolites in schizophrenia (73, 74), future studies should attempt to obtain spectroscopy data from MRI scanners that are able to separate these metabolites. Spectroscopy was done during a resting state, thus the correlations with the BOLD signal obtained during task cannot be interpreted as being causal. Further studies combining fMRI with functional MRS, where changes in neurometabolites are measured during task performance, might provide a more fine grained understanding of the link between metabolites and cognitive processes. Finally, at baseline patients made more errors during the congruent trials of the Stroop task than during the incongruent trials, which is unusual.

## Conclusion

In off-medication patients, we observed an altered relationship between ACC Glx and BOLD response in regions of the salience, including the ACC, and posterior DM networks compared to HC. These results suggest a mechanism whereby alterations in the relationship between cortical glutamate and BOLD response is disrupting the modulation of major neural networks sub serving task-negative rest and task-positive cognitive processes, potentially affecting cognition. While these relationships appear to normalize with treatment in patients, the interpretations of the results are confounded by significant group differences in Glx levels, as well as the variability of the relationship between Glx and BOLD response in HC over time, which may be driven by factors including habituation to task or scanner environment.

## Author contributions

AL was responsible for the study concept and design. AL and DW supervised the study. EC, NK, and AL conducted the statistical analysis and drafted the manuscript. JM and EN conduced additional analyses in response to the review. BG assisted with patient recruitment. All authors contributed to acquisition, analysis, or interpretation of the data and critically reviewed the content of the manuscript for important intellectual content. AL is the guarantor.

### Conflict of interest statement

The funding source had no role in the design and conduct of the study; collection, management, analysis, and interpretation of the data; preparation, review, or approval of the manuscript; and decision to submit the manuscript for publication. Medication for this study was donated to AL by Janssen Pharmaceuticals, Inc. AL received an investigator initiated grant from Janssen Pharmaceuticals, Inc. The remaining authors declare that the research was conducted in the absence of any commercial or financial relationships that could be construed as a potential conflict of interest.
