# Supplementary material for: A Longitudinal Multimodal Neuroimaging Study to Examine Relationships Between Resting State Glutamate and Task Related BOLD Response in Schizophrenia
Source: Front Psychiatry. 2018 Nov 29;9:632. doi: 10.3389/fpsyt.2018.00632 (PMC6281980; doi:10.3389/fpsyt.2018.00632)
Supplement: Supplementary file 1 [file Data_Sheet_1.PDF]

A Longitudinal Multimodal Neuroimaging Study to Examine Relationships between Resting State Glutamate and Task Related BOLD Response in Schizophrenia

Elyse J. Cadena[1], David M.White[1], Nina V. Kraguljac[1], Meredith A. Reid[2], Jose O. Maximo[1], Eric A. Nelson[1], Brian A. Gawronski[1], Adrienne C.Lahti[1]*

***Correspondence: alahti@uab.edu**

Supplement Figure 1

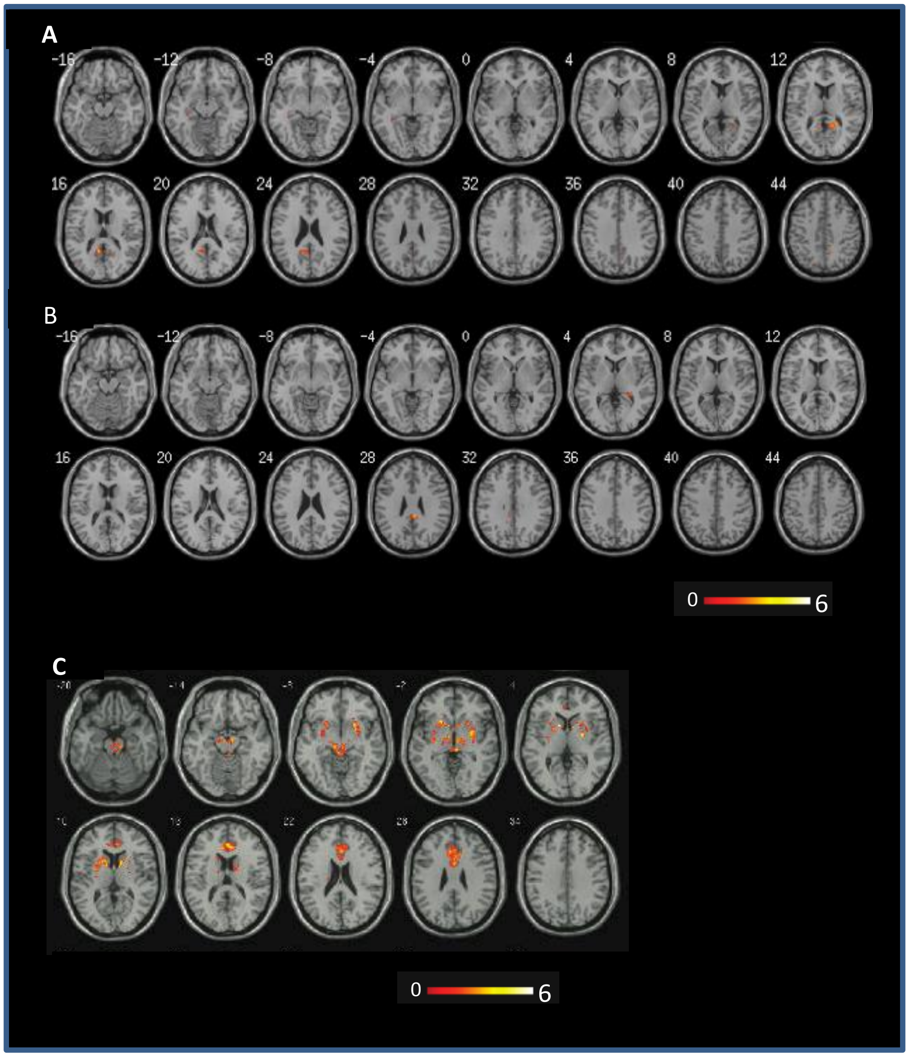

Supplement Figure 1 Legend

Limited deactivation of the posterior default mode network (DMN) during Stroop performance in healthy controls (**A**) and in off-medication patients with schizophrenia (**B**). Analyses were restricted to a mask of the posterior DMN encompassing the bilateral posterior cingulate cortex, precuneus, inferior parietal lobule and hippocampus using a small-volume correction; $p<0.05$. Color bar on bottom indicates t-score. **C.** Significant BOLD activation during Stroop performance in the same healthy controls (Cadena et al., 2018). Analyses were restricted to a mask encompassing the ACC, striatum and midbrain using a small-volume correction; $p<0.05$. ACC: anterior cingulate cortex. Color bar on bottom indicates t-score. Visual inspection of t-score in panel A & B and panel C shows lower t-scores for panel A & B.

Cadena, E. J., White, D. M., Kraguljac, N. V., Reid, M. A., & Lahti, A. C. (2018). Evaluation of fronto-striatal networks during cognitive control in unmedicated patients with schizophrenia and the effect of antipsychotic medication. *NPJ Schizophr*, *4*(1), 8. doi:10.1038/s41537-018-0051-y
