# Supplementary material for: A Longitudinal Multimodal Neuroimaging Study to Examine Relationships Between Resting State Glutamate and Task Related BOLD Response in Schizophrenia
Source: Front Psychiatry. 2018 Nov 29;9:632. doi: 10.3389/fpsyt.2018.00632 (PMC6281980; doi:10.3389/fpsyt.2018.00632)
Supplement: Supplementary file 2 [file Data_Sheet_2.PDF]

A Longitudinal Multimodal Neuroimaging Study to Examine Relationships between Resting State Glutamate and Task Related BOLD Response in Schizophrenia

Elyse J. Cadena[1], David M.White[1], Nina V. Kraguljac[1], Meredith A. Reid[2], Jose O. Maximo[1], Eric A. Nelson[1], Brian A. Gawronski[1], Adrienne C.Lahti[1]*

***Correspondence: alahti@uab.edu**

Supplement Results.

Correlations between Glx levels and behavioral measures

At baseline, HC Glx positively correlated with congruent RT (r= 0.30, p= 0.01). There were no correlations between HC Glx and error commissions. In unmedicated SZ, there were no correlations between Glx and RTs as well as error commissions. At 6 weeks, there were no correlations between Glx and RTs in either HC or SZ. In medicated SZ, but not in HC, Glx positively correlated with incongruent errors (r= 0.22, p= 0.04). There were no significant differences between correlations between SZ patients across time. HC had no significant correlations with committed errors. There were no significant differences between correlations between HC patients across time. There were also no significant differences between correlations between groups across time. SZ Glx levels were not significantly correlated with BPRS total, positive, and negative scores at either baseline or after 6 weeks.
