# Supplementary material for: A Longitudinal Multimodal Neuroimaging Study to Examine Relationships Between Resting State Glutamate and Task Related BOLD Response in Schizophrenia
Source: Front Psychiatry. 2018 Nov 29;9:632. doi: 10.3389/fpsyt.2018.00632 (PMC6281980; doi:10.3389/fpsyt.2018.00632)
Supplement: Supplementary file 3 [file Data_Sheet_3.PDF]

A Longitudinal Multimodal Neuroimaging Study to Examine Relationships between Resting State Glutamate and Task Related BOLD Response in Schizophrenia

Elyse J. Cadena[1], David M.White[1], Nina V. Kraguljac[1], Meredith A. Reid[2], Jose O. Maximo[1], Eric A. Nelson[1], Brian A. Gawronski[1], Adrienne C.Lahti[1]*

***Correspondence:** alahti@uab.edu

Supplement Table 1. Motion Parameters

	SZ 0	SZ 6	HC 0	HC 6
x	-0.00 (0.02)	-0.02 (0.11)	0.00 (0.01)	-0.00 (0.01)
y	0.01 (0.13)	0.02 (0.09)	0.02 (0.04)	-0.00 (0.03)
z	0.05 (0.04)	0.06 (0.09)	0.04 (0.04)	0.04 (0.06)
Pitch	0.00 (0.00)	0.00 (0.00)	0.00 (0.00)	-0.00 (0.00)
Roll	-0.00 (0.00)	-0.00 (0.00)	0.00 (0.00)	-0.00 (0.00)
Yaw	-0.00 (0.00)	-0.00 (0.01)	-0.00 (0.00)	-0.00 (0.00)

Abbreviations: SZ 0, baseline schizophrenia, SZ 6, schizophrenia at week 6; HC 0, baseline healthy control, HC 6, week 6 healthy control
