# Supplementary material for: A Longitudinal Multimodal Neuroimaging Study to Examine Relationships Between Resting State Glutamate and Task Related BOLD Response in Schizophrenia
Source: Front Psychiatry. 2018 Nov 29;9:632. doi: 10.3389/fpsyt.2018.00632 (PMC6281980; doi:10.3389/fpsyt.2018.00632)
Supplement: Supplementary file 4 [file Data_Sheet_4.PDF]

A Longitudinal Multimodal Neuroimaging Study to Examine Relationships between Resting State Glutamate and Task Related BOLD Response in Schizophrenia

Elyse J. Cadena[1], David M.White[1], Nina V. Kraguljac[1], Meredith A. Reid[2], Jose O. Maximo[1], Eric A. Nelson[1], Brian A. Gawronski[1], Adrienne C.Lahti[1]*
***Correspondence: alahti@uab.edu**

Supplement Table 2. Relationship between anterior cingulate cortex (ACC) Glx and the Stroop BOLD signal in schizophrenia and healthy controls at baseline.

Abbreviations: L, left; R, right. ACC, anterior cingulate cortex; PCC, posterior cingulate cortex; Inf. Parietal, inferior parietal cortex; DMN, Default mode network
x, y, z, refer to Montreal Neurological Institute coordinates. Salience network was restricted to ACC and insula. Posterior DMN was restricted to hippocampus, precuneus, inferior parietal gyrus, and PCC ($p<0.05_{SVC}$).

Region	Hemisphere	x, y, z	Voxels	Peak t-value
**Salience Network**				
**HC**				
Cluster 1 (ACC)	R	2, 30 ,14	704	4.36
Cluster 2 (Inula)	R	46, 6, -8	214	3.63
Cluster 3 (Insula)	L	-45, 12, -8	173	3.27
Cluster 4 (Insula)	R	40, 22, 3	100	3.19
Cluster 5 (Insula)	L	-29, 30, 4	77	4.33
Cluster 6 (Insula)	L	-38, -12, 20	71	3.44
Cluster 7 (ACC)	R	5, 20 , 29	59	2.96
**SZ**				
Cluster 1 (ACC)	L	-6, 48, 10	812	4.83
Cluster 2 (Insula)	R	-40, 12, 15	227	4.06
Cluster 3 (Insula)	R	28, 15, -13	75	3.71
Cluster 4 (Insula)	R	43, 18, 2	114	3.77
Cluster 5 (Insula)	R	42, 12, 3	91	3.33
Cluster 6 (Insula)	R	31, 28, 6	78	3.84
Cluster 7 (ACC)	R	15, 45, 7	101	3.42
Cluster 8 (ACC)	R	-2, 22, 26	66	3.00
**Posterior DMN**				
**HC**				
Cluster 1 (Hippocampus)	R	37, -17, -21	91	4.66
Cluster 2 (Hippocampus)	L	-24, -21, -12	86	3.81
Cluster 3 (Hippocampus)	R	24, 33, -1	106	4.75
Cluster 4 (Precuneus)	L	-22, -48, 10	52	3.44
Cluster 5 (Precuneus)	R	12, -40, 6	78	3.39
Cluster 6 (Precuneus)	R	3, -57, 19	222	4.2
Cluster 7 (Precuneus)	R	20, -62, 26	103	4.10
Cluster 8 (Precuneus)	L	-8, -68, 50	1571	4.82
Cluster 9 (Inf. Parietal)	L	-50, -38, 42	62	3.61
Cluster 10 (Inf. Parietal)	R	51, -56, 47	69	3.75
Cluster 11 (Precuneus)	R	15, -50, 51	55	4.38
Cluster 12 (Inf. Parietal)	L	-55, -21, 47	95	3.83
Cluster 13 (Inf. Parietal)	R	46, -41, 53	97	3.39

Cluster 14 (Precuneus)	R	13, -68, 60	58	4.38
Cluster 15 (Precuneus)	L	-16, -42, 66	90	3.54
**SZ**				
Cluster 1 (Hippocampus)	R	39, -12, -21	72	3.87
Cluster 2 (Hippocampus)	L	-30, -14, -19	108	3.77
Cluster 3 (Hippocampus)	R	26, -21, -13	198	4.70
Cluster 4 (Hippocampus)	L	-16, -29, -10	56	3.56
Cluster 5 (Hippocampus)	R	25, -35, 8	82	4.12
Cluster 6 (Hippocampus)	L	-14, -41, 6	114	4.12
Cluster 7 (Precuneus)	R	12, -50, 8	111	4.70
Cluster 8 (PCC)	R	6, -41, 14	116	3.77
Cluster 9 (PCC)	L	-4, -39, 24	59	2.96
Cluster 10 (Precuneus)	R	15, -48, 24	67	3.17
Cluster 11 (Precuneus)	R	3, -63, 38	724	4.14
Cluster 12 (PCC)	R	4, -38, 29	114	3.81
Cluster 13 (Inf. Parietal)	L	-51, -50, 36	75	3.08
Cluster 14 (Inf. Parietal)	L	-26, -56, 39	192	4.73
Cluster 15 (Inf. Parietal)	L	-44, -51, 47	62	4.46
Cluster 16 (Precuneus)	L	-10, -45, 48	63	3.61
Cluster 17 (Inf. Parietal)	L	-34, -56, 47	64	4.00
Cluster 18 (Precuneus)	R	14, -45, 50	302	4.27
