# Supplementary material for: A Longitudinal Multimodal Neuroimaging Study to Examine Relationships Between Resting State Glutamate and Task Related BOLD Response in Schizophrenia
Source: Front Psychiatry. 2018 Nov 29;9:632. doi: 10.3389/fpsyt.2018.00632 (PMC6281980; doi:10.3389/fpsyt.2018.00632)
Supplement: Supplementary file 5 [file Data_Sheet_5.PDF]

A Longitudinal Multimodal Neuroimaging Study to Examine Relationships between Resting State Glutamate and Task Related BOLD Response in Schizophrenia

Elyse J. Cadena[1], David M.White[1], Nina V. Kraguljac[1], Meredith A. Reid[2], Jose O. Maximo[1], Eric A. Nelson[1], Brian A. Gawronski[1], Adrienne C.Lahti[1]*
***Correspondence: alahti@uab.edu**

Supplement Table 3. Relationship between anterior cingulate cortex (ACC) Glx and the Stroop BOLD signal in schizophrenia and healthy controls at week 6.

Abbreviations: L, left; R, right. ACC, anterior cingulate cortex; posterior cingulate cortex (PCC); Inf. Parietal, inferior parietal cortex; DMN, default mode nework
x, y, z, refer to Montreal Neurological Institute coordinates. Salience network was restricted to ACC and insula. Posterior default mode network (DMN) was restricted to the hippocampus, precuneus, inferior parietal gyrus, and PCC ($p<0.05_{SVC}$).

Region	Hemisphere	x, y, z	Voxels	Peak t-value
**Salience Network**				
**HC**				
Cluster 1 (Insula)	L	-26, 27, 9	50	3.96
Cluster 2 (Insula)	R	36, 22, 8	70	3.58
**SZ**				
Cluster 1 (Insula)	R	42, 2, -4	971	4.81
Cluster 2 (Insula)	L	-40, 4, -15	1074	5.19
Cluster 3 (ACC)	L	-9, 51, 2	114	2.82
Cluster 4 (ACC)	R	15, 29, 24	773	4.44
Cluster 5 (ACC)	L	-9, 4, 30	71	3.11
**Posterior DMN**				
**HC**				
Cluster 1 (Inf. Parietal)	L	-34, -36, 36	56	2.64
**SZ**				
Cluster 1 (Hippocampus)	R	38, -9, -15	224	4.96
Cluster 2 (Hippocampus)	L	-24, -31, -1	84	4.03
Cluster 3 (Hippocampus)	R	28, -32, -1	78	3.58
Cluster 4 (Hippocampus)	L	-15, -51, 21	69	3.82
Cluster 5 (Precuneus)	R	4, -70, 40	3860	5.07
Cluster 6 (PCC)	L	-3, -33, 27	129	4.66
Cluster 7 (Inf. Parietal)	R	-52, -22, 39	1172	4.39
Cluster 8 (Inf. Parietal)	R	43, -41, 50	753	4.49
